# Nanofiber Expansion of Umbilical Cord Blood Hematopoietic Stem Cells

**Published:** 2015-12-10

**Authors:** F Eskandari, A Allahverdi, H Nasiri, M Azad, N Kalantari, M Soleimani, H Zare-Zardini

**Affiliations:** 1 Department of Hematology and Blood Banking, Faculty of Medical Science, Tarbiat Modares University, Tehran, Iran.; 2 Hematology-Oncology and Stem cell Transplantation Research Center, Tehran university of Medical Science, Tehran, Iran; 3 Department of Medical Laboratory Sciences, Faculty of Allied Medicine, Qazvin University of Medical Science, Qazvin, Iran.; 4 Hematology and Oncology Research Center, Shahid Sadoughi University Medical Sciences and Health Services, Yazd, Iran

**Keywords:** Umbilical cord blood, Polyethersulfon, Nanofiber scaffold

## Abstract

**Background:**

The aim of this study was the ex vivo expansion of Umbilical Cord Blood hematopoietic stem cells on biocompatible nanofiber scaffolds.

**Materials and Methods:**

CD133+ hematopoietic stem cells were separated from umbilical cord blood using MidiMacs (positive selection) system by means of monocolonal antibody CD133 (microbeads); subsequently, flowcytometry method was done to assess the purity of separated cells. Isolated cells were cultured on plate (2 Dimensional) and fibronectin conjugated polyethersulfon nanofiber scaffold, simultaneously (3 Dimensional). Colony assay test was performed to show colonization ability of expanded cells.

**Results:**

Cell count analysis revealed that expansion of hematopoietic stem cells in 2dimensional (2D) environment was greater than 3dimensional (3D) condition (p= 0.01). Assessment of stem cell- phenotype after expansions was performed by flowcytometric analysis which is showed that the maintenance of CD133 marker in expanded cells in 3 dimensional condition were higher than expanded cells in 2 dimensional condition (p=0.01). Moreover, colony assay test was performed before and after of expansion to show colonization ability of expanded cells both in 3D and 2D culture and results revealed more ability of 3D culture compared with 2D culture (p= 0.03).

**Conclusion:**

The results of current study confirmed that umbilical cord blood CD133+ haematopoietic stem cells are able to expand on fibronectin conjugated polyethersulfon scaffold. These findings indicated that 3D is a proper and valuable cell culture system for hematopoietic stem cells expansion, compared to 2D in invitro situation.

## Introduction

Hematopoietic stem cell transplantation (HSCT) is a therapeutic approach in treatment of hematological and non hematological disorders; nevertheless, finding suitable donors for patients is barrier to use them. Hematopoietic stem cells are the rare progenitor cells found mainly in bone marrow and alternatively in peripheral blood and umbilical cord blood. CD133+ hematopoietic stem cells are generally described by the ability to self-renewal cell division. In healthy condition, these cells produce all different form of blood cells and provide homeostatic maintenance (1-4). Recently, umbilical cord blood (UCB) derived Hematopoietic stem cells served as an attractive alternative source to bone marrow for transplantation because of low incidence of Graft Versus Host Disease (GVHD) and HLA (Human Leukocyte Antigen) mismatching (5-7). However, insufficient numbers of HSCs is still a major constraint in clinical applications (2,8, 9). Ex vivo expansion of stem cells is a proper way to overcome this limitation and beside, it may improve the quality of engraftment. (10). For achievement of purpose, hematopoietic stem cells expanded in suspension culture with a cocktail of cytokines and serum free medium. 

In this situation, HSCs expansion occurs in flasks and cell culture plates which provide 2D (2 Dimensional) culture condition; however, topographical properties of bone marrow microenvironment has not been regarded (11-13). Bone marrow microenvironment, nominated "niche", is a complex network of stromal cells and also, extracellular matrix (ECM), which is able to prepare topographical, mechanical and biochemical signals to regulate stem cell functions such as self-renewal, differentiation, migration and homing (14,15).

Stem cell niche also is a dynamic microenvironment that provides physicochemical and biological conditions for seeding of these subjected cells. Because of the important role of ECM, a lot of interests have been paid to mimic the natural ECM. Electrospinning method has been developed to produce nanofiber scaffolds with the similar characteristics of ECM (16-18). In this mentioned method, many different natural and synthetic materials are used for fabricating scaffolds. Some of natural ECM components such as gelatin, collagen and fibronectin also manipulated to improve the surface structure and characteristic of these scaffolds (19). Polyethersulfone (PES) is a biocompatible and non-biodegradable polymer that is used to produce membrane filtration and hemodialysis (19). These materials include advantages because of its well-defined composition, reproducibility of surface chemistry topography, toxicity profile, and degradation rates. 

The aim of current study was to establish the new 3D culture system by using a specific nanofiber. So, polyethersulfone (PES) polymer was used to produce nanofiber scaffolds because its biocompatibility and suitable cells attachment to growth and cell expansion. Then, the scaffolds were coated with fibronectin which may improve cell adhesion and stability during expansion. Finally, ex-vivo expansion of CD133+ hematopoietic stem cells on 3D and 2D cultures were compared together. 

## Materials and Methods:

**Sample Collection and Preparation**

Human umbilical cord blood units were collected from donors with informed consent from Iranian Blood Transfusion Organization. Three units of collected cord blood were used separately as triplicate condition. At first, mononuclear cells (MNCs) were isolated. Briefly, one umbilical cord blood unit was diluted with hydroxyethyl starch (HES) in ratio of 1:3 to eliminate red blood cells (RBCs). Then, by using Ficoll-HyPaque (Pharmacia-Amersham, Piscataway, density 1.077 g/mL) with centrifuge (eppendorf) at 1200 RPM for 30 minutes at 20^◦^C, diluted sample was separated to some layers. At the end, mononuclear cells (MNCs) was collected and washed twice with phosphate buffer saline (PBS) / EDTA. 

**Isolation of CD133+ Cells**

Isolation of CD133+ cells was done using magnetic cell sorting (MACS) technology (Miltenyi Biotec, CA, USA) according to manufacturer’s instruction. MNCs were incubated with 50 μl blocking solution and 50 μl CD133 micro beads (Miltenyi Biotec, CA, USA) for 60 minute at 4^◦^C. Then, Incubated cells were centrifuged at 1200 RPM for 5 minutes at 4^◦^C and resuspended in PBS buffer. Following that, isolation of CD133+ cells was done by LS column and magnetic field by adding cell suspension into the column. At the end, labeled cells with microbead flash out in 15 ml contained tube. Purity of isolated CD133+ cells was confirmed by flowcytometry analysis (Partec-PAS flowcytometer). 

**Scaffold Supplementation and Surface Modification**

Polyether sulfone (PES) nanofiber with nano-sized diameters was taken from Stem Cell Biology Department, (Stem Cell Technology Research Center, Iran). For modifying surface of PES scaffolds, plasma treatment was performed by a plasma generator of 40 kHz frequency and pure oxygen at 0.4 mbar pressure for 5 min (Diener Electronics, Germany). For fibronectin grafting, plasma treated nanofibers were immersed in 1-ethyl-3-(3-dimethylaminopropyl) carbodiimide/N-hydroxysuccinimide (Merck, Germany) solution (5 mg/ml) for 12 h and rinsed with distilled water. Moreover, scaffolds were immersed in 1 mg/ml fibronectin solution (Nutacon BV, Netherlands) for 12 h. Following that scaffolds were rinsed with distilled water. 

**Cell Seeding and Ex Vivo Culture of the HSCs**

Prior to cell seeding, in order to sterilize, scaffolds were incubated in 70% ethanol overnight and then washed 3 times with PBS. Scaffolds were placed in 48-well polystyrene plates (grainer). Isolated CD133+ cells were seeded on nanofiber scaffolds at a number of 5×104 cells/scaffold. Then, serum free cell culture medium stem span (stem cell technologies, USA) containing 50 ng/ml SCF(Stem Cell Factor), 50 ng /ml FLT-3 ligand and 50 ng/ml TPO (Thrombopoietin) (Gibco, USA) were added to scaffolds and plate were placed in incubator at 37^◦^C and 5% CO2 for 14 days. The medium was changed every 48 hours. Then CD 133+ cells were cultured in 48- well plate as a 2D cell culture condition in same medium as above and the medium was changed every 48 hours. At day 7 and 14, cells were harvested from scaffold and plate, and then counted by Hemacytometer. The viability of cells also was determined by Trypan blue.

**Scanning Electron Microscopy Assay**

To evaluate the growth and morphology of cells, SEM (Scanning Electron Microscopy) imaging was done at day 7 and 14 of culture. The cell-seeded scaffolds were rinsed with PBS and fixed in gelutaraldehyde 2.5% for 1 h. For dehydration, scaffolds were placed in a series of gradient alcohol concentration and then dried.

**Flowcytometry Analysis**

Flowcytometry analysis was performed to determine the expression of CD133. Cells extracted from the fibronectin coated scaffold and 2D culture at defined times (day 7 and 14 of culture). These subjected cells were incubated with phycoerythrin (PE) conjugated CD133 monoclonal antibody (Miltenyi Biotec, Auburn, CA) for 30 minutes in dark space at 4^◦^C. CD133 labeled cells analysis was done by a FACS Caliber flowcytometer (Becton Dickinson, NJ, USA). At the end, all data was analyzed by ciflowgic software.

**Colony assay**

5000 Expanded cells in plate and nanofiber were harvested at day 7 and 14 to compare the ability of colony formation with cells of day 0. Cells were added to 2 ml of methylcellulose medium (H4435, stem cell technology, Canada) plated in 6 well dishes (grainer). After 14 days incubation in 37◦C and 5% CO2, colonies included CFU-GM, BFU-E/CFU-E, and CFU-GEMM (CFU-Mix) were counted.

**Statistical Analysis**

Statistical analysis was performed using SPSS (V.16) software and p < 0.05 was considered significant.

## Results

**Flowcytometry analysis**

**Figure 1:**
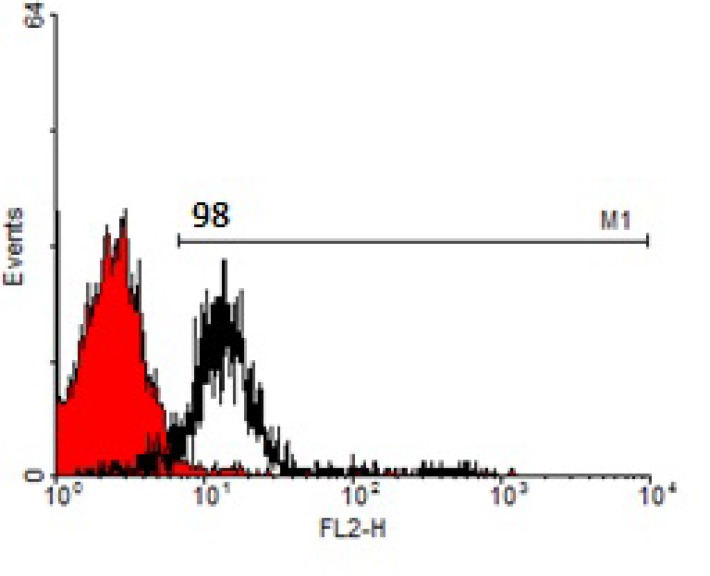
Purity of CD133^+^ cells after isolation

Purity of isolated CD133+ cells was evaluated by flowcytometry method, immediately after isolation and the acquired result was 98%. It was proper enough for continuing the downstream steps as shown in Figure 1.

**Polyethersulfone Nanofiber Surface Modification and Characterization**

**Figure 2:**
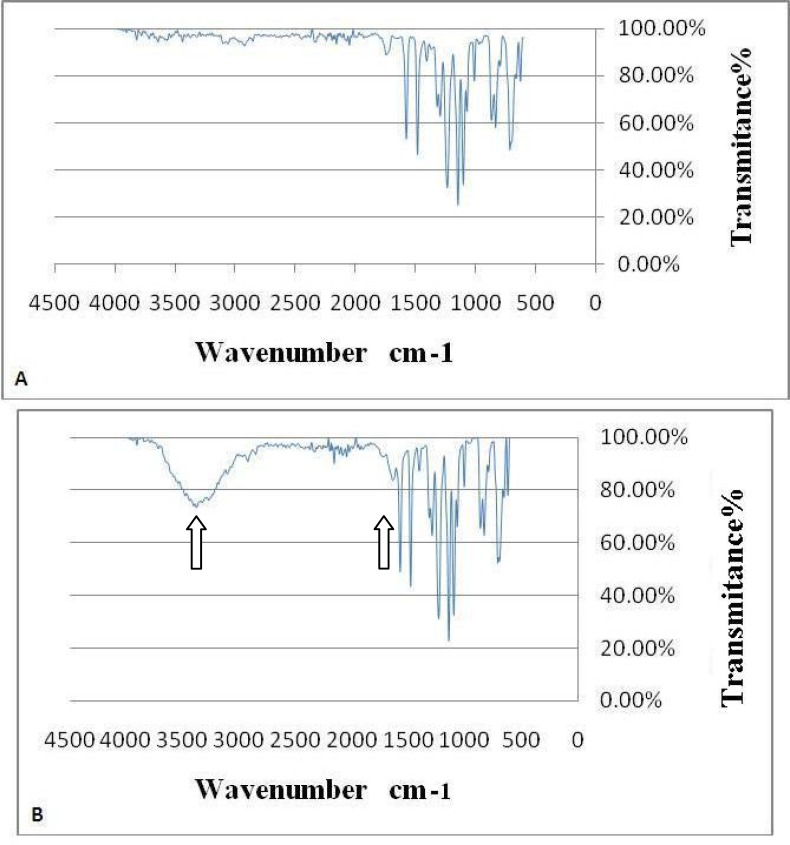
A-PES nanofiber before fibronectin coating B-PES nanofiber scaffold after fibronectin coating

ATR-FTIR spectroscopy was performed to confirm the hydrophility ability and grafting of fibronectin onto the surface of PES nanofibers (Figure 2).New peaks were observed in IR spectrum of PES-fibronectin compared with PES. These were typical characteristic peaks of carboxyl groups (3436 cm-1) related to hydrophilic ability and amide type 1 (1661 cm-1) related to fibronectin.

**Expansion of Cells in 2D and 3D Conditions**

**Figure 3:**
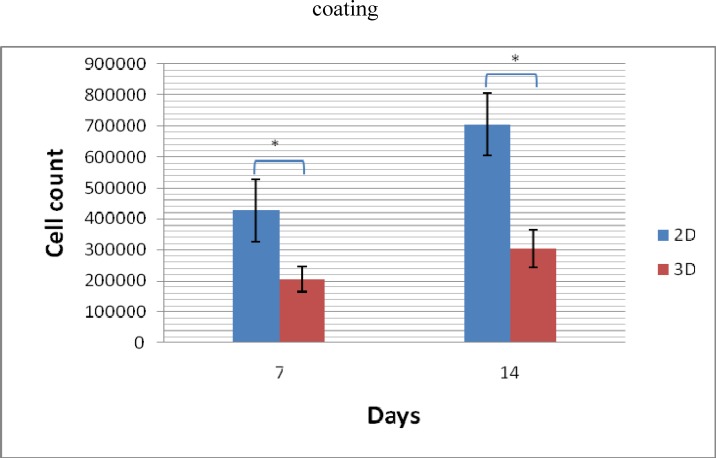
Expansion of CD133^+ ^cells at day 7 and 14 in 2D and 3D environment

CD133+ cells in both 2D and 3D culture condition were expanded for 7 and 14 days (Figure 3). To compare the cell expansion in both media, after 7 and 14 days expansion of cells in serum free culture, subjected cells from every media were counted. Results showed that expansion of cells in 2 dimensional culture was higher compared with cells seeded on Fibronectin coated PES scaffold conditions. The acquired result was statistically significant (p= 0.01).

**Scanning Electron Microscopy and Morphology Assay**

**Figure 4:**
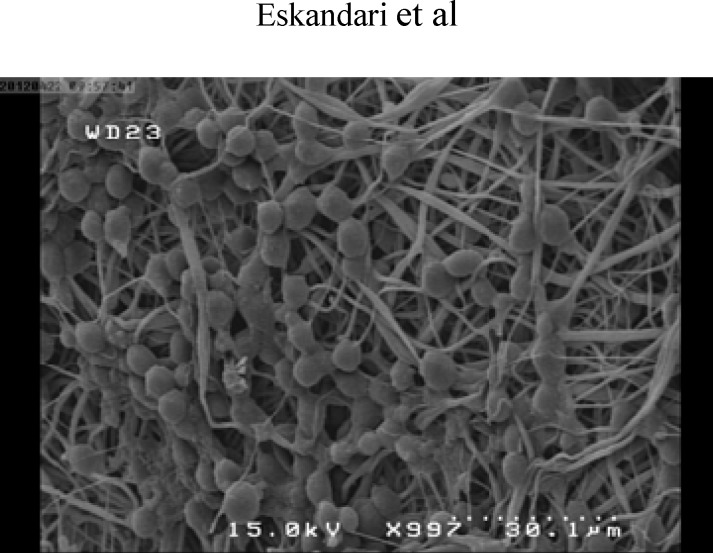
SEM imaging of expanded cells on nanofiber scaffold X400

Evaluation of cellular morphology and its attachment were performed by scanning electron imaging after expansion of cells on nanofiber scaffold. In this part, the result depicted the suitable morphology of evaluated cells (Figure 4).

**Colony Assay**

**Figure 5.**
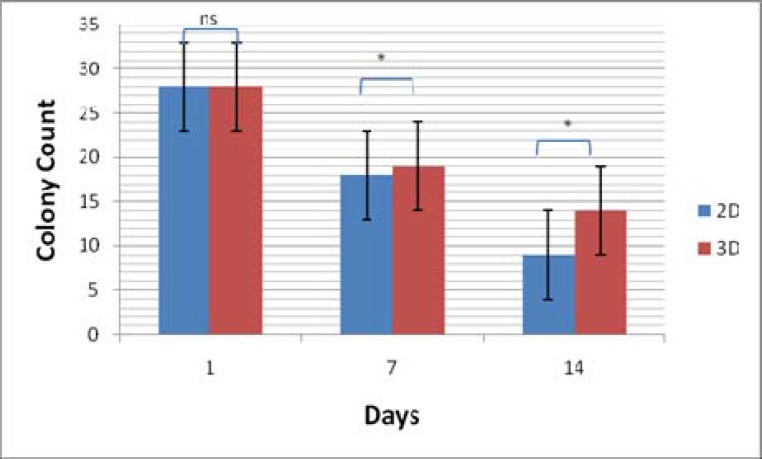
Colony assay test of isolated and expanded CD133+ cells in both 2D and 3D condition

Colony assay was done to evaluate colonogenic potential of expanded cells. For this assay, 5000 of expanded cells from both 2D and 3D culture conditions at day 7 and 14 of expansion harvested and added to 2 ml of methylcellulose medium. The results revealed that cells expanded on 3D nanofiber scaffold yielded higher total colony counts compared with 2D plate condition and both of them were lower than primary isolated cells and these differences were statistically significant (p =0.03)(Figure 5).

**CD133 Expression Analysis**

**Figure 6:**
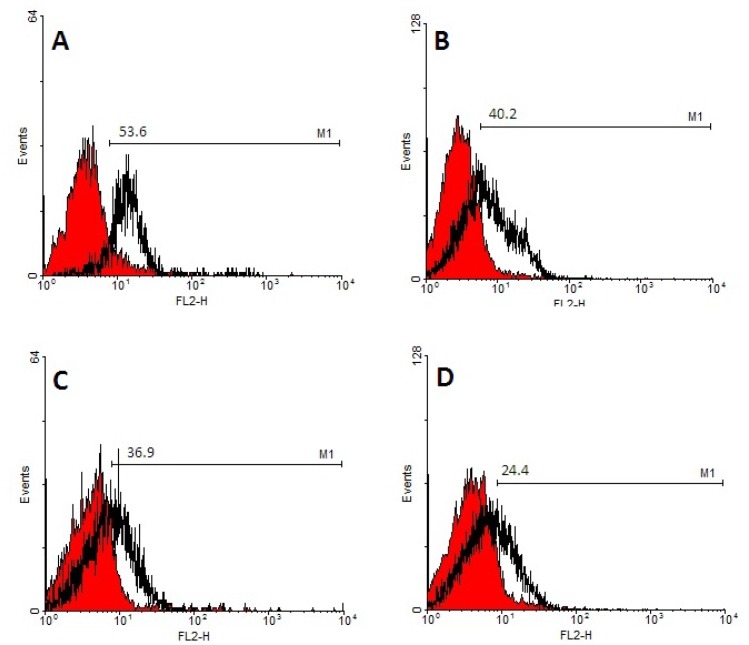
A) Expression of CD 133^+^ cells in 3D microenvironment at day 7 (53.6%) - B) Expression of CD 133^+^ cells in 2D microenvironment at day 7 (40.2%) - C) Expression of CD 133^+^ cells in 3D microenvironment at day 14 (36.9%) - D) Expression of CD 133^+^ cells in 2D microenvironment at day 14(24.4%)

Surface CD133 expression of expanded cells in both 2D and 3D scaffold was evaluated by flowcytometry at day 7 and 14 of culture. Analysis of data revealed that CD133 expression in 2D culture was lower than 3D condition cells and these differences were statistically significant (p= 0.01) (Figure 6).

## Discussion

Hematopoietic stem cell transplantation has been used as a standard treatment for various hematological disorders. Recently, umbilical cord blood hematopoietic stem cells are recommended as a new and alternative source to these cells. Using umbilical cord bloods have many advantages compared with bone marrow and peripheral blood. However, low count of stem cells in a single unit of umbilical cord blood remains as main challenge that hampers using of stem cell. Ex vivo expansion of stem cells can overcome this limitation and improve the quality of engraftment. In conventional culture conditions which performed in plates as 2 dimensional (2D) environments, cell expansion was not considered the importance of cell-cell interaction and stem cell microenvironment cues. Stem cells were resided in a complex network of stromal cells and extracellular matrix (ECM) where provide topographical, mechanical, and biochemical signals that regulate stem cells function such as self-renewal, differentiation, migration, and homing. This network also called niche is a dynamic microenvironment that makes available physicochemical and biological conditions for seeding of cells. It consists of three major components: cell-cell contacts, cell-extracellular matrix (ECM) interactions, and cell-soluble factor interactions. 3D condition is used to increase the surface to volume ratio in order to increase cellular interactions. In 2D cell culture condition, these interactions are insufficient. Because of the important role of these factors in controlling the HS/PC self-renewal and differentiation, it is hypothesized that immobilization of cell-adhesion molecule on substrates can provide a functional substrate that mediated adhesive property. This study used fibronectin conjugated nanofiber scaffold for expanding the CD133+ hematopoietic stem cells. Fibronectin was used due to its important role in attachment and cell surface signaling of stem cells. The obtained results demonstrated that total cell expansion on 3D environment was lower than plate condition as 2D environment. In another study, Feng et al; showed that expansion of CD34+ on Fibronectin-conjugated PET film resulted in significantly higher total cell expansion compared to 2D cell culture (21). Also, Ferreira et al; demonstrated that using fibrin and collagen coated PCL scaffold, with and without co- culture by cord blood mesenchymal stem cell had large number of CD34+ cell compared to 2D cell culture microenvironment (20). In this study, differentiation of expanded cells on 3D culture was lower than those in 2D condition by flowcytometry method and number of CD133+ cells on 3D culture was higher than 2D culture. Ehring B et al; demonstrated that scaffolds conjugated with fibronectin and collagen had more total colonies than 2D cell culture system but this difference was not statistically significant. In this study, in 3D culture system, the total number of colonies was higher than that of 2D cell culture system and results were statically significant. Therefore, these results confirmed that using fibronectin coated nanofiber had the ability to maintain cells in undifferentiated state compared to 2D condition. Moreover, the SEM images prepared direct evidence of morphology and adhesion of HSCs on nanofiber scaffolds. 

## Conclusion

This study indicated that CD133+ hematopoietic stem cells derived from umbilical cord blood can be expanded on fibronectin coated biocompatible nanofiber scaffold. The results revealed that differentiation of stem cells on 3 dimensional (3D) scaffolds were lower than 2D plate culture. On the other hand, acquired results in the current study proved that nanofiber-based ex-vivo expansion technology was able to generate stem cells for potential clinical and therapeutic applications.

## Conflict of interest

The Authors have no conflict of interest.
